# Transient Destabilization of Declarative Memory—Opposing Impact of Physical Exercise or Rest after Encoding in Typically Developing Children and Children with Attention Deficit Hyperactivity Disorder but No Difference after Subsequent Sleep

**DOI:** 10.3390/brainsci12030322

**Published:** 2022-02-27

**Authors:** Manuel Munz, Lioba Baving, Alexander Prehn-Kristensen

**Affiliations:** 1 Central Outpatient Department, Center for Integrative Psychiatry, School of Medicine, Christian-Albrecht University Kiel, 24105 Kiel, Germany; 2 Department of Child and Adolescent Psychiatry and Psychotherapy, Center for Integrative Psychiatry, School of Medicine, Christian-Albrecht University Kiel, 24105 Kiel, Germany; lioba.baving@uksh.de (L.B.); alexander.prehn-kristensen@uksh.de (A.P.-K.); 3 Department of Psychology, MSH Medical School Hamburg, University of Applied Sciences and Medical University, 20457 Hamburg, Germany

**Keywords:** declarative memory, attention deficit hyperactivity disorder, typically developing children, exercise, sleep

## Abstract

Background: Children are especially sensitive to a broad range of influences and show a remarkable capacity for learning. One prominent example is declarative memory, which may be influenced by a variety of factors and is impaired in attention deficit hyperactivity disorder (ADHD). Exercise and sleep, or both combined, might foster declarative memory. Methods: Here, 12 typically developing children (TDC) and 12 age-matched children with ADHD participated in an exercise and rest condition before a night in the sleep laboratory. Declarative memory was encoded before exercise or rest and retrieved before and after a night of sleep. Results: Exercise in TDC but rest in ADHD lead to a transient destabilization of declarative memory, while there were no more differences after a night of sleep. Rapid eye movement (REM) sleep latency was prolonged after exercise in both groups. Conclusions: Exercise leads to opposing effects on immediate declarative memory formation. The factors or contexts that promote or hinder declarative memory formation in children ADHD and TDC differ, and further work is needed to determine the recommendations for declarative learning in children with ADHD.

## 1. Introduction

The formation and long-term storage of declarative memories comprises various processes and may be influenced by multiple antagonistically or synergistically interrelated factors [1]. While the process of initially acquiring a memory trace is commonly labelled “encoding”, the recall of a memory trace from an intermediate or permanent store is named “retrieval”. The transformation from an intermediate to a permanent store is commonly labelled “consolidation”, or if a memory trace has been externally or internally re-activated before, “re-consolidation” [2,3]. When initially encoded, the newly acquired memory will be in intermediate storage and be vulnerable to potential interference. Once consolidated and thus stable and less susceptible to interference or forgetting, this memory trace will be ready for retrieval for many months or even years [4,5]. Among various conditions, both sleep and exercise can support declarative memory consolidation [6]. On a neural level, hippocampal−neocortical interactions have been found to be crucial in memory formation, consolidation, and reconsolidation [5].

There is compelling evidence that sleep benefits declarative memory consolidation more than wakefulness [1], particularly when individuals are told that there will be retrieval of encoded information after an interval of consolidation [7]. This contrast between sleep and wake offline consolidation is more pronounced in children, who express higher levels of slow-wave sleep (SWS) [8,9]. The significance of SWS for declarative memory consolidation, i.e., organizing the redistribution of newly acquired memories from the hippocampus to the cortex [10], has been studied extensively [1]. Although not studied as comprehensively as SWS or sleep in general, physical exercise and, more specifically, cardiovascular exercise have been found to foster memory consolidation or at least protect memories from deterioration when individuals are sleep-deprived [6]. An exercise-induced increase in hippocampal−cortical functional connectivity as measured by functional magnetic resonance imaging [11] has been hypothesized to be a candidate neural mechanism. Furthermore, evidence has been put forward that physical exercise and sleep might synergistically interact to enhance memory consolidation [6].

The developing brain shows a remarkable capacity for plastic changes and is especially sensitive to a wide range of experiences [12]. Children acquire large amounts of non-declarative skills and declarative information while being exposed to a variety of sensory stimuli and an abundance of information, which is a growing factor with omnipresent digital media. On the other hand, physical exercise is highly recommended from a developmental perspective [13] and has been linked to better cognitive performances in school-aged children [14]. However, the every-day environmental or physical factors, which are beneficial or might interact with declarative memory consolidation in children both during wakefulness and sleep, have not been explored so far.

Attention deficit hyperactivity disorder (ADHD) is characterized by developmentally inappropriate levels of inattention, hyperactivity, and impulsivity [15]. Among other neurocognitive impairments, such as working memory deficits or impaired executive functions in general [16,17], deficits in declarative memory consolidation have also been found in ADHD, both in adults [18,19] and in children [20,21]. Declarative memory skills are important for scholastic development and for social interaction [22]. With a prevalence of ADHD of 5–7% [23], these learning deficits are of relevance for one or two or even more children in almost every school class. Declarative memory deficits in ADHD have been hypothesized to be an epiphenomenon of defective prefrontal functioning during (slow wave) sleep [24,25], and we demonstrated previously that this deficit could be compensated for by direct-current stimulation over prefrontal areas [26]. Probably more suitable or usable on a larger scale in terms of treatment, closed-loop acoustic stimulation during SWS enhanced (rewarded) declarative memories in typically developing children (TDC) but not in children with ADHD [27]. Given its positive effect on memory, physical exercise might be a straightforward and inexpensive approach to alleviate memory deficits in persons with ADHD.

We are only beginning to understand the exact impact of physical activity on sleep and memory [6]. Based on the finding of enhanced functional connectivity of areas involved in declarative memory processing [11] and the positive effects of acute aerobic exercise on memory formation in adults [28], we hypothesized that exercise has a positive impact on declarative memory consolidation in children too. With respect to a potential interaction of exercise and sleep, one potential link is the possible enhancement of SWS through (intense) physical exercise, which has been demonstrated in adolescents [29]. Using an intra-individual design, we investigated the impact of daytime physical exercise on declarative memory evolution with the encoding of information before a session of exercise or rest while listening to an audio drama, and followed by a period of wake, with retrieval before and after a night of sleep in TDC and children with ADHD. We hypothesized that intense physical exercise would enhance the wake offline consolidation of declarative memory, increase SWS, and support sleep-dependent consolidation compared to a non-active rest condition in both groups.

## 2. Materials and Methods

### 2.1. Participants

Here, 12 male children diagnosed with ADHD according to DSM-IV-TR [15] (6 with the combined subtype and 6 with the inattentive subtype; mean age 10.7 ± 0.22 years, range 9–11 years) and 12 typically developing boys (mean age 10.2 ± 0.23 years, range 9–11 years) were included in the study. Four of the 12 patients diagnosed with ADHD also fulfilled the criteria for oppositional defiant disorder (ODD). ADHD patients were recruited via the outpatient unit of the Department of Child and Adolescent Psychiatry and Psychotherapy of the Center for Integrative Psychiatry in Kiel, Germany, or by newspaper advertisements, through which healthy control subjects were also recruited. In order to confirm the diagnoses (patients) or exclude any psychopathology (in the typically developing boys), respectively, all children and their parents were interviewed using a German translation of the Revised Schedule for Affective Disorders and Schizophrenia for School-Age Children: Present and Lifetime Version (Kiddie SADS-PL) [30,31]. Additionally, the Child Behavior Checklist (CBCL) [32], a standardized questionnaire as part of the Achenbach System of Empirically Based Assessment (ASEBA), was filled out by parents to assess any psychiatric symptoms in their children. Controls were excluded if they displayed any mental issues. Exclusion criteria for all participants were diagnoses other than ADHD or ODD, any continuous medication other than stimulants for the ADHD group or any medication in the controls, below average intelligence quotient (IQ < 85), as measured by the Culture Fair Intelligence Test20-Revised Version CFT 20-R [33], or profound memory impairment as measured by a figural learning test to assess cerebral dysfunctions (Diagnosticum für Cerebralschädigung, DCS; [34]; cut-off score: 16th percentile of the reference sample). The patients and controls did not differ in age or pubertal stage. For the TDC group, there was a trend to higher scores in the brief estimation of intelligence using CFT-20R, part one. However, memory scores in the DCS were equal between the respective groups. According to self-ratings, all were classified as pre-pubertal (Pubertal Development Scale (PDS)) [35]. One participant in each group was left-handed and all other participants were right-handed, as assessed with the Edinburgh Handedness Inventory [36]. According to the Sleep-Self-Report questionnaire (SSR) [37] (critical score > 24), there was no difference between patients and healthy controls with respect to sleep problems (ADHD: median: 24.5, range: 18–27, M = 23.8, SD = 3.0; healthy controls: median: 21.0, range: 18–27, M = 21.6, SD = 2.4; ADHD vs. controls: *t*(22) = 2.77, *p* = 0.110). However, parental ratings on the Children Sleep Habit Questionnaire ((CSHQ) [37]; critical sore > 41) revealed more sleep problems in children with ADHD than healthy controls, as indicated by a higher sum score in the ADHD group (ADHD: median: 43.0, range: 37–44, M = 42.3, SD = 2.6; healthy controls: median: 40.0, range: 35–45, M = 39.4, SD = 3.3; ADHD vs. controls: *t*(20) = 5.8, *p* = 0.025). Seven of the 12 patients were on methylphenidate; this medication was discontinued 48 h (13 half times) prior to each test session. All participants had to pass a medical examination that consisted of history taking, physical examination, and exercise electrocardiogram (ECG). While there was a trend towards a lower resting heart rate in the control group (69.4+/−2.6 beats per minute (bpm) for controls and 76.2+/−2.7 bpm for ADHD; F(22) = 3.247; *p* = 0.086); systolic blood pressure (F(22) = 0.328; *p* = 0.572) and diastolic blood pressure were equal between the ADHD and the control group (F(22) = 1.253; *p* = 0.275). Although the calculated maximal heart rate (Hfmax [38]) was statistically different between the ADHD individuals and controls (ADHD: 209.8 +/− 0.2 bpm and controls: 210.4 bpm +/− 0.2 bpm; F(22) = 5.254; *p* = 0.032), the mean difference of 0.6 bpm can be considered technically irrelevant as the range of our exercise condition was quite broad (85–90%; see Section 2.2). Power when reaching Hfmax in the exercise ECG was comparable between the groups (ADHD: 125 +/− 5.2 W; controls 122.3 +/− 7.2 W; F(22)= 0.09; *p* = 0.762). All of the participants reached their calculated Hfmax with no symptoms or irregularities in the ECG. Participants’ characteristics are given in Table 1.

### 2.2. Experimental Design and Procedures

**Figure 1:**
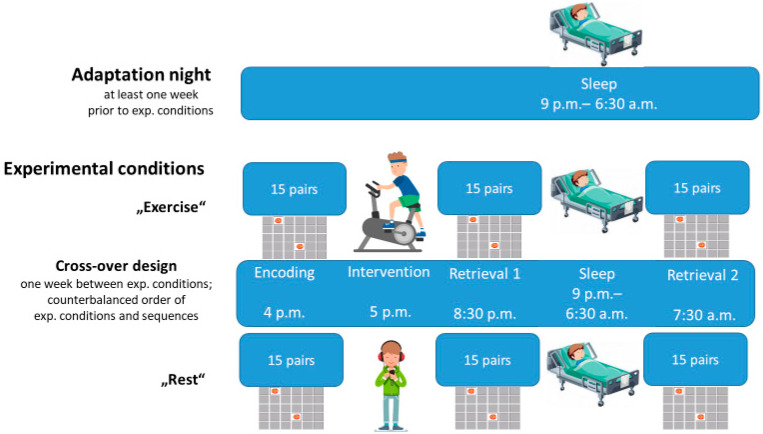
Overall design—timeline of encoding, intervention (exercise and rest), Retrieval 1, sleep, and Retrieval 2 (adopted from [40]).

The study was approved by the ethics committee of the Medical Faculty of the University of Kiel and followed the Declaration of Helsinki. We used a 2 × 2 × 3 factor design with the between-factor GROUP (ADHD vs. controls) and the within factors of CONDITION (exercise vs. rest) and TIME (Encoding, Retrieval 1, and Retrieval 2). All participants underwent two experimental sessions consisting of a declarative memory encoding session (E), followed by an interval of intense exercise in the first experimental night and rest in the second experimental night or vice versa, a retrieval session in the evening (R1), followed by a night of sleep and a second retrieval session (R2) in the morning. The first experimental session was preceded by an adaptation night in order to familiarize participants with the sleep laboratory. The experimental sessions were at least one week apart. Declarative memory was assessed with a computerized 2D object location task based on the game “Concentration” (see below). Intense physical exercise consisted of four 10-min blocks of intense exercise with 85–90% of the calculated Hfmax [39] on an automated ergometer suited for children with resting intervals of at least 5 min between the exercise blocks. For the rest condition, children were seated comfortably and listened to an age-appropriate audio drama under the supervision of one of the experimenters. The procedures are depicted in Figure 1.

### 2.3. Declarative Memory Task

The 2D object-location task has previously been used in adults [41] and in children [27]. During encoding, participants were presented with a series of 15 pairs of cards and were instructed to memorize the respective objects and locations. Hereafter, one by one, the first card of a pair was uncovered and the children were asked to recall the correct location of the corresponding card. The recollection of all 15 pairs made up one trial. Trials were rerun until 9 (60%) of the 15 desired cards were located accurately. During R1 and R2, there was only one trial, irrespective of the number of accurately located cards.

### 2.4. EEG Data Collection

All EEG data were collected in the sleep laboratory of the Center for Integrative Psychiatry in Kiel using a 33-channel somnography device (Somnocreen plus PSG+, Somnomedics, Randesacker, Germany). Electrodes were placed according to the international 10–20 system at the locations F3, F4, C3, Cz, C4, Pz, O1, and O2 referenced to the bridge of the nose with a ground placed at Fpz. EOG was recorded from the lower right and upper left canthi in a diagonal fashion. EEG and EOG were sampled at 256 Hz (band-pass filter: 0.2–35 Hz). EMG was recorded from the chin and from the left and right lower legs at 128 Hz (0.2–150 Hz). During the adaptation night, nasal air flow, nasal air pressure, and chest motion were monitored with a thermistor and a piezoelectric band, respectively. The raw data were visually scored [42] by a trained EEG technician. REM and NREM sleep stages were defined according to AASM. Time in bed was calculated as the interval between lights out and lights on. Total sleep time was calculated as the minutes spent asleep during time in bed. Sleep efficiency was calculated as the percentage of time asleep compared to the time spent in bed.

### 2.5. Statistical Analysis

Statistical analysis was performed with IBM SPSS Statistics, version 25, for Windows. With respect to declarative memory performance, correct card pairs were analyzed by an analysis of variance (ANOVA) for repeated measurements with the between factor GROUP (ADHD vs. controls) and the within factors EXERCISE (exercise vs. rest) and TIME (E vs. R1 vs. R2). In addition, working memory was analyzed as a cognitive control parameter using ANOVA for repeated measurements with the between factor GROUP (ADHD vs. controls) and the within factors EXERCISE (exercise vs. rest) and SESSION (E vs. R1 vs. R2). Differences in single means were tested by paired *t*-tests.

## 3. Results

### 3.1. Sleep Parameters

ANOVA for repeated measurements with the within-factor CONDITION (exercise vs. rest) and the between-factor GROUP (ADHD vs. controls) revealed no main effect of CONDITION, no main effect of GROUP, and no GROUP × CONDITION interaction for the duration of sleep stages (REM, N1, N2, and N3), total sleep time, or sleep efficiency (*p* > 0.05). The polysomnographic results are depicted in Table 1. However, comparable to prior reports [40], there was a main effect of CONDITION for REM latency (F(1.21) = 24.6, *p* < 0.001) with no effect of GROUP (F(1.21) = 0.05, *p* = 0.943) or GROUP × CONDITION interaction (F(1.21) = 0.3, *p* = 0.56). Paired *t*-tests revealed that REM latency—corresponding to the duration of the first sleep cycle—was profoundly prolonged in the exercise condition both in ADHD individuals (exercise: M = 154.9 min, SEM = 12.6; rest: M = 110.3 min, SEM = 15.7; *p* = 0.01) and in the controls (exercise: M = 150.2 min, SEM = 12.4; rest: M = 104.4 min, SEM = 9.8; *p* = 0.002). Sleep data are summarized in Table 2.

### 3.2. Declarative Memory

In the 2 × 3 × 2 ANOVA with the within factors CONDITION (exercise vs. rest) and SESSION (E, R1, R2) and the between factor GROUP (ADHD vs. controls), there was no main effect of CONDITION (F(1.21) = 0.01, *p* = 0.81), but a main effect of SESSION (F(2.34) = 26.02, *p* < 0.001). Moreover, there was trend to a GROUP effect (F(1.21) = 3.69, *p* = 0.068).

**Figure 2:**
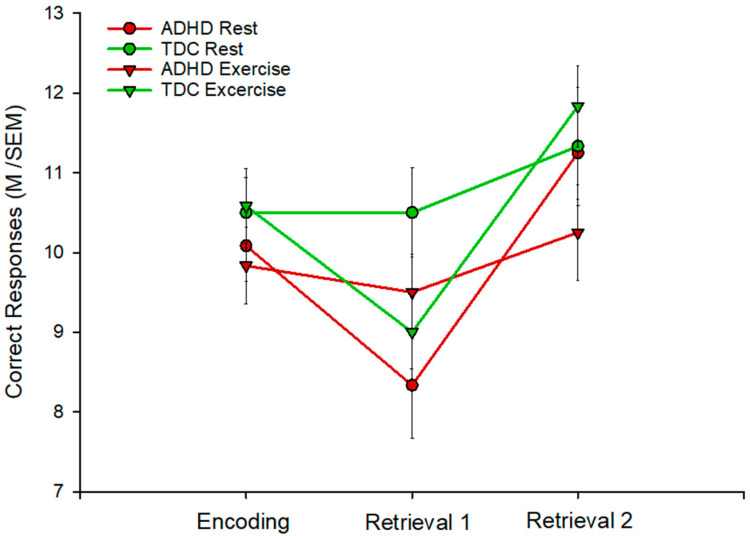
Results of the declarative memory task: mean (M) and standard error of mean (SEM) number of correctly recalled card pairs in children with attention deficit hyperactivity disorder (ADHD) and typically developing children (TDC) during the EXERCISE (red) and REST (green) conditions.

There was no CONDITION × SESSION (F(1.21) = 0.19, *p* = 0.824), SESSION × GROUP (F(1.21) = 0.02, *p* = 0.982), or CONDITION × GROUP interaction (F(1.21) = 0.18, *p* = 0.675). However, there was a GROUP × CONDITION × SESSION interaction (F(1.21) = 7.18, *p* = 0.002). The interaction was decomposed with *t*-tests. This indicated that, with respect to the REST condition and starting from a comparable number of word pairs in both groups (ADHD: M = 10.08; SEM = 0.50; controls: M = 10.5; SEM = 0.44; *t*(11) = 1.07; *p* = 0.298), there was a decline in card pairs recalled in R1 in the ADHD group (M = 8.33, SEM = 0.66; *p* = 0.008), which was not true for the controls (M = 10.5; SEM = 0.56; *p* = 1). After a night of sleep, the number of accurately located card pairs in R2 was again equal between the groups (ADHD: M = 11.25, SEM = 0.48; controls: M = 11.33; SEM = 0.74; *t*(11) = 0.09; *p* = 0.982), with a rise in the ADHD group overnight (*p* < 0.001) that was not there in the controls (*p* = 0.175). In the EXERCISE condition, this pattern was exactly reversed: starting from equal numbers of card pairs (ADHD: M = 9.83; SEM = 0.30; controls: M = 10.58; SEM = 0.47; *t*(11) = 1.35; *p* = 0.19), there was a decline from E to R1 in the control group (M = 9.0, SEM = 0.46; *p* = 0.001), which was not present in the ADHD group (M = 9.5, SEM = 0.54; *p* = 0.59). There was an overnight gain in the control group (M = 11.83, SEM = 0.50; *p* < 0.0001), while there was a trend to an overnight gain in ADHD individuals (M = 10.25, SEM = 0.60; *p* = 0.0687). The results of the declarative memory task are given in Table 3 and Figure 2.

### 3.3. Working Memory

With respect to our working memory control task, there was a main effect of SESSION (F(2.34) = 18.19, *p* = 0.002) and a main effect of GROUP (F(2.34) = 5.00, *p* = 0.037), but no main effect of CONDITION (F(2.34) = 2.52, *p* = 0.128). There was no GROUP × SESSION (F(2.34) = 0.69, *p* = 0.509), no GROUP × CONDITION (F(2.34) = 0.43, *p* = 0.521), and no GROUP × CONDITION × SESSION (F(2.34) = 1.87, *p* = 0.167) interactions.

## 4. Discussion

We investigated the effect of acute physical exercise with immediate retrieval after an interval filled with daytime wakefulness and delayed retrieval after a night of sleep. In typically developing children, we found reduced accuracy in a 2D object-location task after exercise compared to passive listening to an audio drama after immediate wake time consolidation, whereas there was no more difference during delayed retrieval after a night of sleep. In a group of age-matched children diagnosed with ADHD, we found the inverse pattern, with reduced accuracy of memory retrieval after audio drama listening while there was no difference after exercise. Again, after a night of sleep, there was no difference between the exercise and the rest condition. Substantially prolonged REM-latency, which we reported for typically developing children [40], was true for children with ADHD too. Consistent with the literature [16,17], working memory was superior in typically developing children compared to children with ADHD and was better in the morning session compared to the afternoon and evening, but there was no influence of exercise or rest.

In contrast to our hypothesis that we were expecting physical exercise to induce plasticity and declarative memory consolidation during wake and sleep, we observed that it did not generally support declarative memory consolidation in typically developing children or children with ADHD. For typically developing children, this inferior immediate retrieval after physical exercise does not necessarily mean that they performed worse than after rest. One might interpret this decline as being an induction of plasticity, as indicated by transient destabilization [4] while receptive for reconsolidation, followed by beneficial sleep-associated consolidation. Employing a resting session after encoding produced no such decline or a difference in performance from the exercise condition the next morning.

The most prominent finding in our experiment is the inverse pattern in children with ADHD compared to typically developing children. While exercise might lead to transient destabilization of declarative memory in healthy children, this was true for children with ADHD in our rest condition, while exercise produced no such effect. We assume that acute exercise might foster plasticity in typically developing children, leading to the attenuation of declarative memory, while a contrasting activity, such as listening to an audio drama, shows a similar transient destabilization in children with ADHD. The transient destabilization might indicate that exercise has a more stimulating effect on the brain than rest, while for untreated children with ADHD, passive auditory input might represent a stressful stimulus and exercise is perceived as more usual or “normal”. In light of hyperkinetic symptoms and an accumulation of physical activity from earlier in the day before encoding and physical exercise, a “saturation” or passing of a maximum of an inversed u-shape of plasticity as a function of activity following the Yerkes−Dodson law [43] might serve as another explanation for why there is no such acutely destabilizing effect in ADHD after exercise. Given the comparable performances in the morning sessions of both groups and in both conditions, our results are not conclusive regarding whether physical activity or rest has an acute beneficial effect on declarative memory formation in the long run.

Unlike word lists [21,41], retrieval in our 2D object location paradigm entails recall and active reconsolidation through repetition in one session. Although probably less appealing for children, using simple word lists to test declarative memory with multiple retrieval sessions might have been more accurate to understand the pure effect of exercise or rest, respectively, on immediate wake time and delayed sleep-dependent memory consolidation. With our paradigm, in which we hypothesized that there could be a transiently destabilizing effect through exercise in healthy children and through audio drama listening in ADHD, reconsolidation during R1 might have been more effective and thus could have compensated for a probably less favorable effect. Along the same line and considering a possible destabilizing effect because of the above-mentioned activities, interfering declarative content as a further extension of the design vs. reconsolidation could have answered the question if plasticity was induced [4]. In addition, our overnight experiment, comprising the within-factor “exercise” and the between factor “ADHD”, lacks a daytime control condition with exclusively wake consolidation intervals, controlling for off-line consolidation not related to sleep and circadian effects. Because of its benefits through physical exercise, executive functioning might have influenced immediate recall during R1 too. Covering only one aspect of executive functioning, our working memory data indicate no such effect of exercise.

Of note, we observed a substantial overnight gain of declarative memory from R1 to R2 in ADHD children in the rest condition (*p* < 0.001) and no gain or loss in the exercise condition (*p* = 0.59). This is in sharp contrast to our previous findings in children with ADHD [26], where, when using the same declarative memory task, there was a significant decline in memory performance from pre-sleep encoding to retrieval in the morning (*p* = 0.001). With the results of our experiment, we cannot claim that the overnight gain with audio drama before immediate recall and reconsolidation and equal performance in R1 (evening) and R2 (morning) was amenable to the respective activities. However, considering better overnight consolidation in both conditions in children with ADHD, reconsolidation by repetition of declarative memory content proved beneficial for sleep-dependent consolidation in ADHD. Moreover, the direct comparison of the ADHD groups from the present (10.7 +/− 0.2 years) and the previous study (12.1 +/− 1.4 years) should be regarded with caution because of the age differences.

## 5. Conclusions

In conclusion, we found opposing acute effects of physical exercise and audio drama listening on the immediate declarative memory retrieval in typically developing children and children with ADHD, with differences being no longer present after a night of sleep. Based on our results, we cannot conclusively argue for one or the other activity as being supportive of sleep-dependent declarative memory formation in children with or without ADHD. However, confirming previous findings that the developing brain is highly sensitive to different environmental conditions and stimuli [12], we can carefully conclude that this applies to declarative memory formation too. Future studies on the initial formation of declarative memories should systematically target or control for influences like physical exercise, physical rest, acoustic sensory input, wake or sleep, time of day, and developmental age. Larger studies with larger samples and allowing for a systematic variation of possible influences with reasonable statistical power are needed to understand the complex nature of declarative memory during development. Our study also suggests that the contexts that foster or hinder declarative memory in children with ADHD are different from the contexts that are beneficial or unfavorable for declarative memory formation in typically developing children. With regard to the opposing effects of audio drama and exercise on immediate retrieval, we suggest that the effective promotion of cognitive skills (e.g., exercise having a positive effect on immediate recall of declarative memory in ADHD) might differ among children with and without ADHD. Given the high prevalence of ADHD (5–7%) [23] and poorer academic performance in children with ADHD [44], future studies to explore different aspects of learning, such as declarative and procedural memory, rule learning, or acquiring social skills, should not only consider contexts such as exercise or rest, but also account for differences of typically developing children and children with ADHD by extending the design with an ADHD group.

## Figures and Tables

**Table 1:** Sample characteristics.

	ADHD M(SEM)	TDC M(SEM)	F(22)	*p*
Age	10.7 (0.22)	10.2 (0.23)	2.068	0.153
estimation of IQ (CFT-20R, part one)	104.1 (2.0)	111.4 (3.1)	3.820	0.064
DCS	53.8 (6.5)	51.0 (7.0)	0.090	0.767
PDS	3.33 (0.3)	3.08 (0.1)	0.529	0.475
**ASEBA (T-values)**				
withdrawn/depressed	60.25 (2.1)	52.8 (1.3)	9.480	0.005
somatic complaints	56.4 (2.4)	56.5 (2.6)	0.001	0.981
anxious/depressed	59.6 (2.4)	56.3 (1.8)	1.166	0.292
social problems	59.3 (2.9)	51.7 (1.0)	5.947	0.023
thought problems	54.0 (2.6)	53.0 (2.0)	0.094	0.763
attention problems	67.4 (2.2)	51.0 (0.9)	47,124	<0.0001
rule breaking behavior	61.5 (2.6)	51.1 (0.6)	15,046	>0.001
aggressive behavior	62.3 (2.9)	50.1 (0.7)	14,865	>0.001
internalizing problems	60.2 (2.4)	54.1 (2.2)	3.396	0.079
externalizing problems	61.4 (2.9)	43.9 (2.2)	22,713	<0.0001
Total	64.0 (2.4)	48.4 (1.8)	27,007	<0.0001
**Sleep**				
SSR	23.4 (0.9)	21.6 (0.7)	2.776	0.110
CSQH	43.3 (0.7)	39.4 (1.0)	5.801	0.025
**physical examination**				
resting heart rate (bpm)	76.2 (2.7)	69.4 (2.6)	3.247	0.086
systolic blood pressure (mmHg)	112.1 (1.1)	111.3 (0.9)	0.328	0.572
diastolic blood pressure (mmHg)	80.8 (1.0)	79.6 (0.4)	1.253	0.275
calculated maximal heart rate (bpm)	209.8 (0.22)	210.4 (0.2)	5.254	0.032
max_power (W)	125 (5.2)	122.3 (7.2)	0.094	0.762

Means (M) and standard error of mean (SEM) of sample characteristics. CFT—culture fair test; ASEBA—Achenbach System of Empirically Based Assessment; DCS—Diagnostikum für Cerebralschädigung; PDS—pubertal development scale; SSR—sleep-self-report; CSHQ—Children’s sleep habits questionnaire; bpm—beats per minute; mmHg—millimeters of mercury; W—watts.

**Table 2:** Sleep data: means (M) and standard error of mean (SEM) of total sleep time (TST); sleep efficiency (SE); sleep onset latency (SOL); REM onset latency (REM-L); non-REM sleep stages N1, N2, and N3; total non-REM sleep (NREM); and total REM sleep (REM). * *p* < 0.001.

	ADHD		TDC							
	Exercise	Rest	Exercise	Rest	Main Effect of Condition	*p*	Main Effect of Group	*p*	Group × Condition Interaction	*p*
	M (SEM)	M (SEM)	M (SEM)	M (SEM)	F(1.22)		F(1.21)		F(1.21)	
TST (min)	496.1 (6.7)	506.6 (11.3)	484.6 (7.9)	499.4 (13.8)	2.08	0.164	00.62	0.439	0.06	0.831
SE (%)	91.2 (0.9)	89.8 (1.4)	86.8 (1.9)	89.9 (2.1)	0.28	0.602	10.50	0.235	2.21	0.152
SOL (min)	23.1 (3.2)	26.7 (3.9)	38.0 (7.4)	32.2 (10.2)	0.03	0.866	10.80	0.194	0.55	0.468
REM-L(min)	154.9 (12.6)	110.3 (15.7)	103.2 (9.4)	159.8 (13.9)	24.57	<0.001 *	00.005	0.943	0.35	0.563
WASO (min)	24.0 (4.4)	29.7 (5.8)	34.7 (7.8)	22.6 (6.3)	0.28	0.604	00.08	0.779	2.08	0.164
N1 (min)	44.7 (4.8)	34.5 (2.7)	44.7 (4.8)	43.2 (3.9)	3.68	0.07	10.14	0.298	1.77	0.198
N2 (min)	234.6 (8.3)	233.8 (10.2)	226.8 (7.5)	230.0 (9.3)	0.03	0.872	00.32	0.578	0.08	0.786
N3 (min)	119.7 (7.8)	128.5 (6.8)	120.0 (6.0)	127.0 (6.8)	2.61	0.121	00.005	0.944	0.03	0.862
NREM (min)	399.0 (6.3)	396.8 (6.5)	391.8 (8.5)	400.2 (10.4)	0.14	0.717	00.04	0.844	0.10	0.535
REM (min)	97.1 (5.7)	109.9 (5.9)	92.8 (4.2)	99.2 (8.3)	4.00	0.058	00.46	0.507	1.02	0.324

**Table 3:** Means (M) and standard error of mean (SEM) of correctly recalled word pairs during Encoding (E—afternoon), Retrieval 1 (R1—evening), and Retrieval 2 (R2—morning).

	REST			EXERCISE		
	E	R1	R2	E	R1	R2
**Adhd** M(SEM)	10.1 (0.50)	8.3 (0.66) *	11.3 (0.48)	9.8 (0.30)	9.5 (0.54)	10.3 (0.60)
**Tdc** M(SEM)	10.5 (0.44)	10.5 (0.56)	11.3 (0.74)	10.6 (0.46)	9.00 (0.46) **	11.8 (0.50)

* ADHD after REST: R1 < E, R2 (*p* < 0.01); ** TDC after EXERCISE: R1 < E, R2 (*p* < 0.01).

## Data Availability

Due to ethical, legal, or privacy issues, data should not be shared.
